# Co‐Design of a Unified, International Aphasia Awareness Campaign

**DOI:** 10.1111/hex.70658

**Published:** 2026-04-15

**Authors:** Claire Bennington, Ciara Shiggins, Jytte Isaksen, Emma Beesley, Kim Beesley, Jean‐Marie Annoni, Jude Czerenkowski, Carly Davey, Natalie Douglas, Alexia Kountouri, Caitlin Longman, Apoorva Paurinak, Sarah Scott, Kathryn Shelley, Sarah J. Wallace

**Affiliations:** ^1^ Queensland Aphasia Research Centre, School of Health and Rehabilitation Sciences The University of Queensland Brisbane QLD Australia; ^2^ Surgical Treatment and Rehabilitation Service (STARS) Education and Research Alliance The University of Queensland and Metro North Health Brisbane QLD Australia; ^3^ Centre of Research Excellence in Aphasia Recovery and Rehabilitation Australia; ^4^ School of Health Sciences the University of East Anglia Norwich UK; ^5^ Department of Language and Culture University of Southern Denmark Odense Denmark; ^6^ Neurorehabilitation Research and Knowledge Centre, Rigshospitalet Copenhagen Denmark; ^7^ Research Advisor with Lived Experience of Aphasia Brisbane Australia; ^8^ Association Internationale Aphasie Brussels Belgium; ^9^ Aphasie Suisse Lucerne Switzerland; ^10^ Stroke Foundation Melbourne Australia; ^11^ Consumer Representative UK; ^12^ Stroke Association London UK; ^13^ University of Louisiana at Lafayette Lafayette Louisiana USA; ^14^ Consumer Representative Cyprus; ^15^ Cyprus Stroke Association Limassol Cyprus; ^16^ Department of Psychological Services and Health Strathclyde University Glasgow UK; ^17^ World Federation of Neurorehabilitation North Shields UK; ^18^ Aphasia Center of West Texas Midland Texas USA; ^19^ Aphasia Access Alexandria Virginia USA

**Keywords:** aphasia, awareness, campaign, co‐design, involvement, PPI, stroke

## Abstract

**Introduction:**

Poor aphasia awareness is an international problem. Previous aphasia awareness campaigns have lacked coordination across countries and have not routinely involved people with the diversity of skills needed to design a campaign. We aimed to co‐design a unified, international aphasia awareness campaign.

**Methods:**

An international, multi‐stakeholder co‐design project was conducted, using online iterative workshops. People with lived experience of aphasia (*n* = 3), representatives of aphasia, stroke, and rehabilitation organisations (*n* = 3), and experts in media, marketing, health promotion, and implementation science (*n* = 3), collaborated across six rounds of co‐design workshops. Three meetings were held per round (18 meetings in total) to accommodate time zones and availability. Workshop topics included: (1) individual experience, motivations, and preferences for collaborating, (2) campaign outcome and target audience, (3) campaign message, (4) campaign format and design, (5) campaign call/s to action and operationalisation, and (6) final review and celebration. Workshops were conducted using Zoom videoconferencing and were recorded. After each meeting, key discussion points, decisions, and outcomes were summarised and synthesised. Framework and Thematic analyses were used to identify themes across all meetings. Involvement was reported per the Guidance for Reporting Involvement of Patients and the Public (GRIPP) 2 short form.

**Results:**

The desired outcome of the campaign was: ‘One day the world's population will understand aphasia and all people with aphasia will be treated with respect and kindness.’ All other campaign elements aligned with this outcome, including the target audience (general public), messages (clarifying what aphasia is/is not and its impact), call/s to action (how to respond to a person with aphasia, where to learn more, and ways to engage), and a tag line (Recognise, Respond, Respect). An operationalisation plan was also co‐developed.

**Conclusion:**

A blueprint for a culturally responsive, unified, international aphasia awareness campaign and strategy for operationalisation was co‐designed. Future directions include co‐developing multi‐lingual and culturally sensitive campaign materials, implementation, and scale‐up.

**Patient or Public Contribution:**

Two research advisors with lived experience of aphasia contributed to project conceptualisation, workshop planning and facilitation, analysis, and dissemination. Additionally, the co‐design team involved three members with lived experience of aphasia and three representatives from key organisations. All contributed to the manuscript preparation.

**Acronyms**
FK: Flesch Kinkaid
GRIPP 2: Guidance for Reporting Involvement of Patients and the Public 2
PAOLI: People with Aphasia and Other Layperson Involvement
PLWA: people living with aphasia
PPA: primary progressive aphasia
PPI: patient and public involvement
QR: quick response
TV: television
UK: United Kingdom
USA: United States of America

## 1 Introduction

Aphasia is an acquired language impairment caused by brain injury (most commonly stroke), which disrupts communication and impacts participation in daily life [1]. Internationally, aphasia awareness is low. This is despite aphasia being a relatively common condition and increased awareness being a priority for the global aphasia community [2, 3, 4, 5]. Many organisations have attempted to raise awareness through campaigns and other activities [3]. Despite considerable efforts, rates of aphasia awareness have not changed substantially over two decades [6, 7]. In March 2022, it was announced that the actor Bruce Willis had been diagnosed with aphasia [8]. This put an unprecedented spotlight on aphasia with news outlets and the public around the world suddenly wanting to know more. The National Aphasia Association (NAA) undertook their 2022 Aphasia Awareness Survey in the United States of America (USA) in the immediate aftermath of the Bruce Willis announcement and found a significant increase in both awareness of the word ‘aphasia’ and basic knowledge about aphasia [9]. However, ten months later, a further announcement from the Willis family provided a more specific diagnosis of frontotemporal dementia [10]. Many news agencies published stories explaining that Willis’ aphasia had ‘progressed into frontotemporal dementia’. An unprecedented leap in understanding of aphasia was quickly undone. More people than ever had heard of aphasia, but many now believed it to be a condition that could result in dementia [11]. Consequently, the persistent problem of poor aphasia awareness has been exacerbated by widespread misinformation leading to an urgent need for a global evidence‐based public education campaign, designed in partnership with people with aphasia.

There are many possible reasons for the lack of aphasia awareness campaign success. Simmons‐Mackie et al. [7] highlighted that campaigns have not had a unified, compelling message; have lacked coordination across organisations and countries; tend to target people who are already aware of aphasia; are not evidence based; have had insufficient inclusion of people with lived experience in their design and no evaluation of their impact. An international survey of 105 people living with aphasia (PLWA) and 306 people who work with people with aphasia (workers) identified a lack of clarity about the meaning of ‘aphasia awareness’, and, therefore what the corresponding campaign aims, goals, and priorities should be. PLWA and workers considered aphasia awareness to mean: (1) knowing how to communicate with a person with aphasia, (2) understanding the condition of aphasia, and (3) educating people about aphasia. Both groups wanted others to know: (1) what helps people with aphasia to communicate, (2) that aphasia does not affect intelligence, and (3) the impacts of aphasia. PLWA and workers reported additional barriers to raising awareness: PLWA reported their communication disability and associated lack of confidence, stigma, and the ‘hidden’ nature of aphasia were key barriers to their participation in, and the success of aphasia awareness campaigns. Workers cited a lack of time, funds (the majority of campaigns received no funding), resources, and skills in marketing, communications, and impact measurement, and a perceived lack of interest amongst the general public [3].

In contrast, people living and working with aphasia have reported facilitators to raising awareness: PLWA reported that campaign success was facilitated by including personal aphasia stories and featuring well‐known individuals with aphasia in campaigns, and by positive public attitudes toward people with aphasia. Similarly, workers highlighted the importance of including PLWA (including celebrities) in campaigns and sharing their stories. Obtaining designated campaign funding, having support from employers, larger organisations and charities, and including professionals with specific campaign expertise, such as marketing skills, were also important [3].

There is an increasing expectation and desire to involve people with lived experience of health conditions (consumers) in research in roles other than as participants [12]. Internationally, many different terms are used to describe this approach, including Co‐design, Patient and Public Involvement (PPI) and Consumer Involvement [13]. The National Institute for Health Research in the UK (NIHR INVOLVE [14]) defines PPI as:

By public involvement we mean research being carried out ‘with’ or ‘by’ members of the public rather than ‘to’, ‘about’ or ‘for’ them.

(NIHR INVOLVE, n.d.)

Consumers can be involved at different levels from being informed, consulted, working collaboratively, through to initiating and leading research (Irish Health Research Forum, 2014). Undertaking research with people with lived experience facilitates building knowledge using their experiential perspective and aims to be more equitable, inclusive, and relevant by responding to community needs. The involvement of end‐users in design and development has been shown to result in meaningful and useful solutions that are likely to be implemented [15]. This theory has been successfully tested in other areas of aphasia research [16, 17]. PPI is a fundamental guiding principle in this project, which draws on the identified barriers and facilitators and consensus‐based priorities for a unified aphasia awareness campaign [18] and aimed to co‐design: (1) a blueprint for a unified international aphasia awareness campaign, and (2) a plan to operationalise the campaign.

## 2 Methods

### 2.1 Patient and Public Involvement (PPI)

Two research advisors with lived experience of aphasia (one person with aphasia (author EB) and one family member (author KB) were members of the research team (lead author CB, and her advisory team CS, JI, EB, KB, and SJW) and involved from the outset of the project; in planning and design, identification of co‐designers, workshop co‐facilitation, and dissemination of results. The involvement of advisors with lived experience was mapped against the PAOLI (People with Aphasia and Other Layperson Involvement) Framework, a consensus‐based guide for patient involvement in aphasia research [19]; see Supporting Information S1: File 1). Additionally, the co‐design team involved three members with lived experience of aphasia and three representatives of key consumer organisations (see ‘Co‐designers’ section). Their involvement is reported per the Guidance for Reporting Involvement of Patients and the Public (GRIPP) 2 shortform [20] (see Supporting Information S2: File 2).

### 2.2 Design

‘Co‐design’ is a collaborative approach that sits within the banner of PPI [13], in which people from different backgrounds, with different expertise, who will be impacted by the outcome of the project, are involved in the design process [17].

### 2.3 Co‐Designers

A matrix of desired characteristics was devised to support the selection of co‐designers. Diversity in age, biological sex, country of residence, first language, lived experience of aphasia, and required professional expertise was sought. Potential candidates were short‐listed from: (1) expressions of interest from preceding studies [3, 18]; and through (2) direct contact with individuals with a known interest in aphasia awareness, representatives of relevant national and international consumer organisations, and people with specific specialist skills relevant to co‐designing an international awareness campaign including media, marketing, health promotion, and implementation science expertise. Voluntary involvement of co‐researchers is essential, and they must be able to understand the full extent of their commitment when they agree to be involved [21]. When people with aphasia are involved, research processes need to be accessible and inclusive [22], using available tools [23, 24, 25]. Therefore, aphasia‐friendly (communication‐accessible) project information was sent via email. Co‐designers had the opportunity to ask questions about the project and/or their involvement before agreeing to be involved. All co‐designers were invited as collaborative partners and, as such, were considered co‐researchers. All but two people who were invited to join the team accepted. Those who declined had pre‐existing commitments that prevented participation. The final co‐design team comprised three co‐designers with lived experience of aphasia (two people with aphasia and one family member), three representatives of national and international aphasia/stroke/neurorehabilitation organisations, three representatives with expertise in media and marketing, health promotion, and implementation science, and the main research team. The research team (who worked across the whole programme of research [26] comprised international speech pathologists and researchers, and two advisors with lived experience of aphasia. Many team members had multiple relevant roles; e.g., our representative with media and marketing expertise was also a family member of a person with aphasia, co‐founder of an aphasia centre, and co‐founder of a professional aphasia membership association (Aphasia Access; USA). The combined co‐design team is described in Table 1.

**Table 1:** Co‐design team characteristics.

Team member	Role on team and professional background (at time of project)	Nationality	Country of residence	First language
**Core research team**				
C.B.	**PhD student** Speech pathologist Representative of the Australian Aphasia Association (Deputy Chair)	British and Australian	Australia	English
J.I.	**Associate advisor, researcher** Speech pathologist **Representative of a country where English is not the first language**	Danish	Denmark	Danish
C.S.	**Associate advisor, researcher** Speech pathologist Representative of the Australian Aphasia Association (Board member)	Irish	Australia	English
E.B.	**Advisor with lived experience** (person with aphasia)	Australian	Australia	English
K.B.	**Advisor with lived experience** (family member) Representative of the Australian Aphasia Association (Secretary)	Australian	Australia	English
S.J.W.	**Principal advisor, researcher** Speech pathologist	Australian	Australia	English
**Co‐design team**				
S.S.	**Person with aphasia** **Passionate advocate for raising awareness of aphasia**	British	UK	English
A.K.	**Person with aphasia** **Representative of a country where English is not the first language** Stroke Ambassador, Cyprus Stroke Association	Cypriot	Cyprus	Greek
C.D	**Family member of a person with aphasia** **Passionate advocate for raising awareness of aphasia on social media** Representative of a national stroke organisation (Stroke Association) (UK)	British	UK	English
J‐M.A.	**Representative of a national aphasia association (Aphasie Suisse)** **Representative of an international aphasia association (the Association Internationale Aphasie)** **Representative of a country where English is not the first language** Active Emeritus Professor of neurology	Swiss	Switzerland	French/Italian
A.P.	**Representative of the World Federation of Neurorehabilitation** **Representative of a country where English is not the first language** Neurologist	Indian	India	Hindi
C.L.	**Representative of a national stroke organisation (Stroke Association) (UK)** Speech and language therapist	South African	UK	English
K.S.	**Representative with media and marketing expertise** Family member of a person with aphasia Co‐founder of an aphasia centre Co‐founder of a professional aphasia membership association (Aphasia Access) (USA)	American	USA	English
N.D.	**Representative with implementation science expertise** Researcher Speech‐language pathologist	American	USA	English
J.C.	**Representative with health promotion experience** Representative of a national stroke organisation, Stroke Foundation (Australia) Social worker	Australian	Australia	English

*Note:* Primary roles on the project in bold.

### 2.4 Reflexive Statement

The authors acknowledge that our positionality as researchers, clinicians, and advocates shaped the co‐design process. In particular, our commitment to aphasia‐friendly practices required continual reflection on how power, expertise, and accessibility were managed within the team. This reflexivity ensured that the voices of people with lived experience were not only included but also actively influenced decision‐making throughout the project.

### 2.5 Procedures

**Figure 1:**
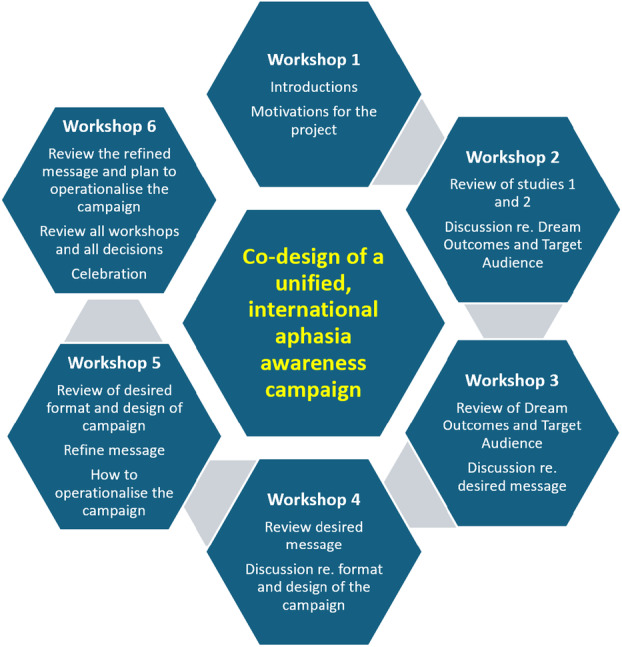
Overview of the co‐design workshop rounds.

Online workshops were held using Zoom (a videoconferencing platform) and were recorded. Workshops used the structure described in Shiggins et al. [17] and were adapted to meet the specific aims of this project. The focus and aims of the workshops are outlined in Figure 1 and detailed in Supporting Informaiton S3: File 3.

**Figure 2:**
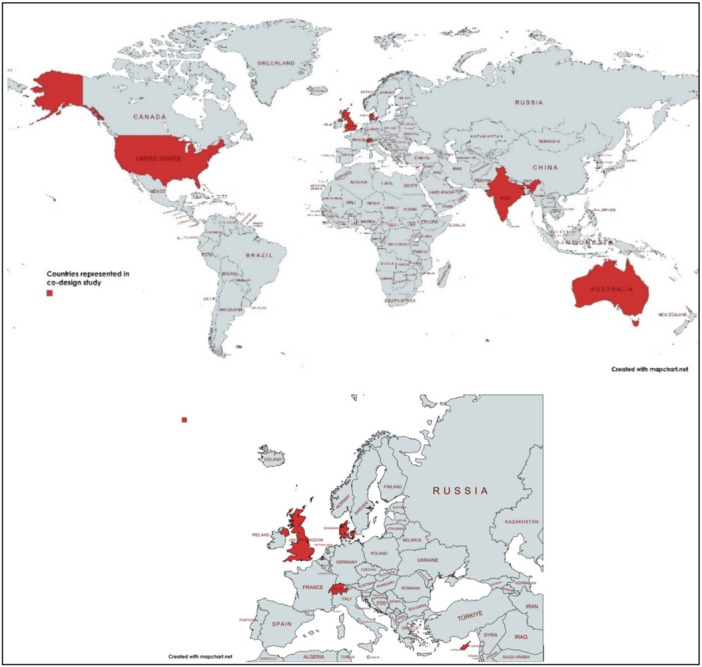
Countries represented by the co‐design team.

**Online workshops.** Six rounds of online workshops were held over 6 months (August 2023 to February 2024). The combined co‐design team was located across seven countries (Australia, Cyprus, Denmark, India, Switzerland, the United Kingdom (UK), and the United States of America (USA); see Figure 2) and eight different time zones.

To manage diverse time zones, three meeting times were offered for each round of workshops (total meetings = 18). Co‐designers could choose to attend any meeting time. Up to 2 h were allocated for each workshop, with scheduled breaks and additional breaks taken as needed. Facilitators regularly checked in with co‐designers about their fatigue levels and reminded them they could request breaks at any time. Supported communication strategies [23] were used throughout, including aphasia‐friendly agendas and presentation slides with simplified language, large text, increased white space, and images to support comprehension [27, 28, 29]. Facilitators summarised key points verbally, allowed additional time for responses, and used visual supports to scaffold discussion. See Supporting Information S4 and S5: Files 4 and 5, which illustrate the communication supports used and how breaks were scheduled.

The roles, responsibilities, and expectations of team members, and the boundaries of the project, were clearly defined as per Shiggins et al. [17], to maximise transparency, accountability, power sharing, and to avoid tokenism and harm (e.g., wasting contributors’ time and effort). This also allowed the opportunity to understand and champion the skills of contributors, and for all co‐design team members to understand each other's experience and skills. Principles of respect, inclusion, and accessibility (NIHR: INVOLVE, n.d.) were maintained throughout the project through aphasia‐inclusive processes [30] and individualised adaptations and accommodations were made to help all co‐designers engage in the process.

The co‐design workshops were facilitated by the research team. CB (based in Melbourne, Australia) chaired every meeting, and other members provided communication and technology support and took notes. An aphasia‐friendly PowerPoint presentation guided each workshop, ensuring consistency across meetings (see Supporting Information S5: File 5). The slides became the basis for accessible meeting minutes, which were shared with all team members. CB kept field notes and reviewed Zoom transcripts, synthesising discussions from each round to present at the start of the next workshop. This summary review was crucial for consistency, continuity, confirmation of ideas, and generating new insights in the iterative co‐design process. Disagreements were approached as productive tensions that enriched the work rather than as obstacles to overcome. Differences of opinion were worked through collaboratively until consensus or an acceptable compromise was reached, with the perspectives of people with lived experience taking precedence where resolution was not possible.

### 2.6 Analysis

#### 2.6.1 During the Workshops

Information (discussion, decisions, outcomes) was collated from each meeting, then synthesised and summarised across the round, and reviewed and refined in the subsequent round.

#### 2.6.2 Following the Workshops

Following the workshops, the discussion was coded using a two‐step process. First, a framework analysis was conducted [31] using the key campaign elements (Desired Outcome, Target Audience, Key Messages, Calls to Action, Tagline, Format and Design of the Campaign and Plan to Operationalise the Campaign) as an a priori coding framework. All meeting minutes, workshop summaries, video recordings, and field notes were reviewed by CB, and information relating to each element was deductively coded. This coding was reviewed by CS, JI, and SJW. Where interpretations differed, the researchers returned to the original recordings to examine the context and refine the coding. Second, codes were analysed inductively by CB using reflexive thematic analysis [32] to identify patterns within and across the campaign elements. Emerging themes were discussed with and reviewed by the research team CS, JI, EB, KB, and SJW.

### 2.7 Credibility

Multiple strategies were used to enhance credibility, transparency, and inclusivity.

#### 2.7.1 Prolonged Engagement and Iterative Design

The research team engaged with co‐designers over a 6‐month period across six rounds of workshops. This sustained interaction enabled the development of trust, mutual understanding, and the co‐construction of knowledge. The iterative nature of the workshops allowed for reflection, refinement, and continuity across rounds.

#### 2.7.2 Triangulation of Ideas and Perspectives

Ideas were drawn from multiple sources, including meeting minutes, field notes, video recordings and transcripts.

#### 2.7.3 Member Checking and Collaborative Validation

Aphasia‐friendly minutes of each workshop round were shared with all co‐designers for review and feedback in the next round. This process enabled member checking and ensured that the outcomes accurately reflected the discussions and decisions of the co‐design team.

#### 2.7.4 Detailed Description and Audit Trail

The co‐design process, including the selection of co‐designers, procedures, workshop structure, and analysis, has been described to support transparency and transferability. The aphasia‐friendly minutes, field notes, video recordings and transcripts, emails to the team, and supervision notes comprise a detailed audit trail.

#### 2.7.5 Ethical and Inclusive Practice

Aphasia‐inclusive strategies were embedded throughout, including the use of aphasia‐friendly materials, supported conversation techniques, and flexible attendance options. These adaptations were essential for equitable involvement and minimising barriers to contribution.

## 3 Results

A total of 18 workshops (1.5–2 h per workshop, totalling 35 h) were convened. Themes are presented narratively per campaign element with illustrative quotes.

### 3.1 Desired Outcome

The desired outcome related to the following themes: (1) normalising the word aphasia ‐ people recognise the word ‘aphasia’ and know what aphasia is and is not; (2) understanding the human experience of aphasia and its enormous impact on a person's life; (3) knowing how to help a person with aphasia including how to support their communication; (4) providing a way to connect to information and services that are already available; (5) understanding the journey of life with aphasia; (6) providing a message of hope; (7) increasing donations and funding for aphasia services and research; (8) positioning aphasia as a social justice issue where communication rights are being violated, or people are not having access to communication. The team debated whether these themes were best suited to an initial campaign or a subsequent campaign. For example, normalising the word aphasia and understanding its impact were considered priorities for an initial campaign, whereas aphasia as a social justice issue should come later, once the word ‘aphasia’ was more widely understood.

All team members agreed with the Dream Outcome, prioritised in Bennington et al. [18]: ‘One day the world's population will understand aphasia and all people with aphasia will be treated with respect and kindness’. CL commented:

I think that that's such a lovely outcome because it unpicks a few key things for me: There's the understanding of aphasia. Understanding for me is a little bit beyond awareness. So, it's not just that they know the term aphasia. It's that they understand it. Then I interpreted that ‘will be treated with kindness,’ that is when people know the impact and how to support.

One of our co‐designers with aphasia, (SS) felt ‘It is also important to know what they [the general public] can do to help us. So, you know, writing down words, maybe, or pictures, or not talking too fast, or there's lots of things that would help as well.’ SS summed up the importance of people knowing how to recognise aphasia and support communication with this quote:

I think it will will be really nice that all of us that have aphasia here can just go out and kind of live our lives not being so worried about just talking to strangers, or going to doctors’ appointments, or going to you know hairdressers’ appointments, or, you know, just the we are the same person before the stroke and after. And yes, we've got a disability, but it's we can still live our lives and be happy.

### 3.2 Target Audience

The team discussed a range of possible target audiences including: (1) the general public/everyone; (2) those who need to know e.g., people with aphasia and their family members and friends; (3) healthcare workers; (4) women aged 40‐60 years old, as they frequently have carer responsibilities; (5) different generations including generations Z and Alpha; (6) specific groups, such as emergency responders and community service providers, e.g., shop workers, hairdressers, café and restaurant workers, and post office and bank staff.

After considering the desired outcome above, the team determined that the target audience for this campaign should be the general public. Across groups, the team felt this audience should be targeted as the general public does not know about aphasia and this audience can be the hardest to reach. It was also thought that a campaign targeting the general public would have a ‘spillover effect’ to other important audiences, including family members and healthcare professionals. Speech pathologists on the team felt that people with aphasia and their family members should receive tailored aphasia education as part of their rehabilitation by speech pathologists and this group should not need to rely on an awareness campaign to learn more about aphasia. The team also felt that health professionals working with patient populations who may experience aphasia should learn about aphasia in their training and ongoing professional development activities, rather than through an awareness campaign.

### 3.3 Key Messages

The discussion themes around the message included: (1) the complexity and variability of aphasia; (2) whether the campaign should apply to aphasia of any aetiology; (3) what information to include/exclude in the campaign; (4) why should people care; (5) how to describe what aphasia is and is not; (6) how to describe the impact of aphasia; (7) whether or not to include some communication strategies; (8) the need for a positive message; (9) links to more information and resources.

The key themes for the message stemmed from the desired outcome and specifically from what the general public would need to know to understand aphasia and how to treat people with aphasia with respect and kindness.

To understand aphasia, the team felt it was important for the general public to know what aphasia is and is not, and to have a sense of the impact of aphasia on a person's life. Of particular importance to our co‐designers living with aphasia was the message that their intelligence is preserved. Our family member (CD), whose mother initially had severe aphasia said: ‘That's my mum's number one thing, if she starts to feel condescended, she says, ‘smart, smart, lady’’. Our co‐designers with aphasia agreed, as illustrated by this quote from SS: ‘We're still intelligent, I think people, just maybe your speech is affected that you're stupid but we're not.’ Lengthy debates occurred around how to describe aphasia, how it masks a person's competence, and its impact. In addition, it was felt these descriptions had to be in succinct terms suitable for a range of campaign materials, including shorter videos. With regard to describing aphasia, the key debates were around terminology: (1) language or communication; (2) problem, difficulty, disorder, challenge, disruption, impairment, or disability; (3) competence, intellect, intelligence, ability to think/thinking normally, capacity, smart, wisdom, cognitive, reasoning, knowledge; (4) description of identity: still the same person inside/I'm still me, I can still do things, versus, being a new version of themselves. Great attention was given to the words used and their frequency, meaning, and readability. The team also felt that there was stigma around certain words such as competence and capacity, as they had legal implications. After considerable discussion across workshops 3‐6, the team settled on the terms ‘communication,’ ‘difficulty’ and ‘intelligence’ believing these would be most meaningful to a general audience. The most important features to highlight were that aphasia can affect talking, understanding speech, reading, writing, and using numbers, but not intelligence, and that aphasia presents differently for everyone. The team felt it was important to say aphasia is caused by a brain injury and that stroke was the most common cause.

With regard to describing the impact of aphasia, the team wanted a compelling message to ‘grab attention,’ stressing the central role communication plays in human life and how the ability to communicate is needed for almost everything we do. Therefore, aphasia affects every part of life (relationships, friendships, work, finances, education, social participation, day‐to‐day activities, such as shopping and banking, leisure activities) and as a result, can lead to social isolation, depression and be very frustrating.

Further detailed debate occurred around how to treat a person with aphasia with respect and kindness and whether this was part of the message or a call to action. The general feeling was that this required highlighting some key communication strategies for the general public to be aware of if they encountered a person with aphasia. This was deemed the behaviour change component of the campaign. It was considered important to stress how using a few simple communication strategies could change a person's life. Therefore, this response was regarded as a call to action and will be elucidated in the next section.

### 3.4 Calls to Action

Having one or more ‘call to action’ was debated. Early in the discussions, some members felt a singular call to action to *learn more* about aphasia (e.g., by clicking a link or scanning a QR code) was sufficient. Others felt that it was important to engage the audience in an action, such as helping to spread the message or experiencing what it is like to have aphasia. Much discussion related to which, of the many aphasia communication support strategies, to include. The team debated using the TALK acronym, which stands for Time, Ask, Listen, Keep trying, but felt this would not translate to all languages for the intended global audience.

The team settled on having three calls to action. The first was how to respond to a person with aphasia in a manner that shows respect and kindness. The key strategies for the general public were: TALK to THEM; Speak SLOWER and CLEARLY; Use SIMPLE words and SHORT PHRASES; Give TIME and LISTEN; DON'T INTERRUPT and KEEP TRYING. A second call to action was to *learn more* (about aphasia and communication strategies) by scanning a QR code leading to further information or a website. A third call to action was an engagement piece to help consolidate the message. Options included: (a) ‘Tell someone else about aphasia because I can't,’ (b) Like and share social media posts, (to help spread the message) and (c) An aphasia challenge, such as ‘Try to order a cup of coffee without speaking’ or an aphasia simulation to provide others with a sense of what it is like to live with aphasia.

### 3.5 Tagline

The team discussed having an easy‐to‐remember tag line for the campaign. Based on all the discussions, they came up with *Recognise, Respond, Respect*, which linked to the outcome, message, and calls to action.

### 3.6 Format and Design of the Campaign

Discussions about the format and design related to the following themes: (1) type, format and style of campaign assets, e.g., social media reel, picture with some text, video, infographic; (2) length of assets; (3) communication channels, e.g., TV, radio, print, social media (Facebook, Instagram, TikTok, X, YouTube, WhatsApp), website; (4) inclusion of real people vs animations; (5) branding (e.g., same colour and logo); (6) inclusive imagery (e.g., of all ethnicities, genders, ages, and abilities); (7) cultural sensitivity; (8) messaging format, e.g., simple, aphasia‐friendly, visual; (9) with or without sound (text or subtitles, voiceover, music).

The team agreed that a suite of downloadable, digital campaign assets (materials), including animated videos of different lengths (e.g., 10 s, 15 s, 30 s) and infographics were needed. These could then be launched across all social media platforms and traditional media outlets. Animations were considered a good option as these could be easily adapted to different cultural and linguistic settings. The team felt that it was essential to use the most common and simplest language terms for the campaign, striving to make the message aphasia‐friendly, as this would make it accessible to the widest possible audience, including those with lower literacy levels. Having aphasia‐friendly text/subtitles was considered important to support accessibility and make the campaign accessible with and without sound. The team wanted all campaign assets to have the same branding (colours and logo), and to be representative of all ethnicities, genders, ages, and abilities. The team felt strongly that all characters in animations and infographics should look human, but there were some mixed views about whether the person with aphasia should be in a wheelchair. The majority felt the wheelchair (and visible disability) might distract the audience from the main message about aphasia, but others did not want people with aphasia who are in wheelchairs to feel they were not represented. It was suggested that photographs of real people of different ages, ethnicities and abilities could be useful for ‘still’ assets. Other ideas included: a website for the campaign; an international symbol for aphasia; an optional badge for people with aphasia to wear explaining that they have aphasia, what aphasia is, and some simple things others can do to help; a wallet card explaining aphasia and some tips to support communication for people with aphasia to use if they wish; a smart phone app which was language and country specific.

**Figure 3:**
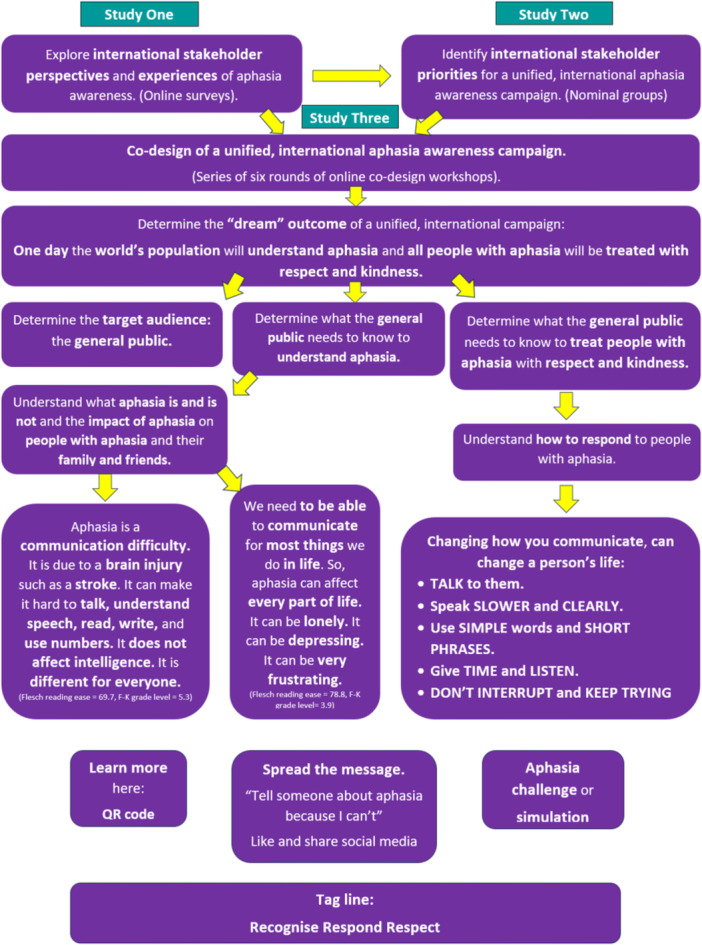
Outcomes of the iterative co‐design decision‐making process for the campaign.

The outcomes of the iterative co‐design decision‐making processes for the campaign are illustrated in Figure 3.

### 3.7 Plan to Operationalise the Campaign

The discussions around how to operationalise the campaign related to the following themes; (1) scale of the campaign including how many countries to involve; (2) language for the campaign: English only vs a multilingual campaign; (3) funding; (4) translations; (5) how and where campaign assets would be kept; (6) distribution model; (7) launching the campaign; (8) evaluating the campaign.

Initially, there was some tension in the group about keeping the campaign to English‐speaking countries with more established aphasia networks, as this felt more manageable, versus trying to reach as many countries as possible. Team members from countries where English is not the first language were very keen that other countries and languages be included, as they felt they are already disadvantaged by not being able to easily share aphasia awareness materials developed in English. The team also acknowledged that most ‘English‐speaking countries’ are multi‐cultural and multi‐lingual, and that an English‐only campaign would be less likely to reach people from other cultures. The team acknowledged that it would be preferable to reach diverse audiences. Therefore, a global, multi‐lingual campaign was agreed on.

The team discussed the need for finance or in‐kind contributions to create and distribute campaign assets. The media and marketing advisor talked about the Ad Council in the USA, which runs campaigns they select for free, but she was not aware of similar models beyond the USA. Other considerations included applying for grants and/or philanthropic funding, seeking opportunities for pro bono support, e.g., from advertising agencies and corporate social responsibility schemes. Our co‐designer from India explained that the Indian Government mandates TV Channels keep a small percentage of airtime for free ads for what is deemed ‘Public Interest.’ The team also discussed how using many social media platforms would be free for sharing posts, but funding would be needed for paid advertising slots or to boost posts.

**Figure 4:**
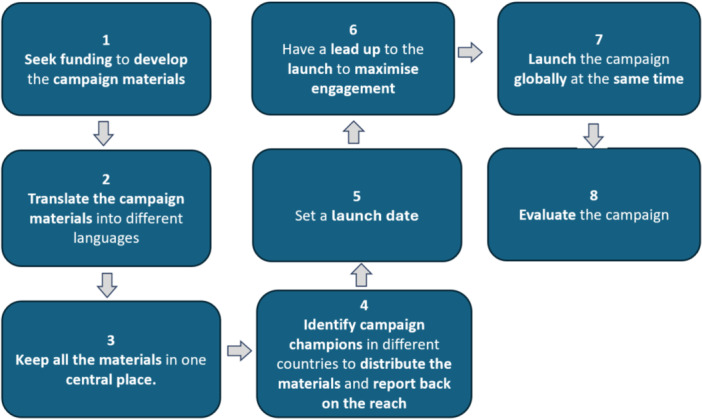
Overview of the plan to operationalise the campaign.

The team discussed and developed a multi‐step strategy to operationalise the campaign, which would be coordinated by an international research team including people with lived experience of aphasia. These steps included: (1) obtaining funding to engage a professional media company to develop the campaign assets with input from consumers to ensure accessibility; (2) arranging translation of campaign materials into as many languages as possible by first language speakers, with verification through a back translation process; (3) organising campaign assets by language and country in centralised digital folders; (4) forming partnerships with key organisations and identifying campaign champions who would distribute assets in their country and report back on reach; (5) setting a co‐ordinated launch date with a lead‐up or countdown; (6) launching the campaign globally at the same time across media channels; (7) measuring the global reach (by tracking social media reach, engagement, and shares, and monitoring website downloads of materials in each country) and evaluating the campaign (by collecting feedback from partner organisations and participants). Figure 4 provides an overview of this operational plan.

Expected outcomes include improved public knowledge, skills and attitudes about aphasia. This means knowing what aphasia is (and is not), the impacts of aphasia, and key communication strategies to use with PLWA. In turn, this may reduce stigma for PLWA and improve communication practices in community and healthcare settings.

## 4 Discussion

An international team, including representatives from non‐English speaking countries, co‐designed a blueprint for a unified international aphasia awareness campaign and a plan to operationalise it. This work reflects a growing international body of inclusive, multi‐lingual work in aphasiology (e.g. [33, 34]). The process was informed by the expertise of our co‐designers, previous studies from within this programme of research [3, 18] and the wider aphasia literature (e.g. [4, 5, 7, 17, 35]). Determining the desired outcome provided the foundation for designing the whole campaign (see Figure 3). It also gave rise to the Tag Line: Recognise, Respond, Respect.

The desired outcome and a key message for the campaign was assisting the general public to understand ‘what aphasia is.’ However, designing this element of the campaign was difficult due to the lack of agreement in the aphasia community as to how to define aphasia [35]. A sticking point for Berg et al. [35] was whether or not aphasia was due to a focal lesion, and this was discussed in this work too. Existing definitions may be too complex for short and compelling campaign assets. For example [36], define aphasia as:

an acquired selective impairment of language modalities and functions resulting from a focal brain lesion in the language dominant hemisphere that affects the person's communicative and social functioning and quality of life and the quality of life of his or her relatives and caregivers.

This definition is 44 words long and has a Flesch Reading Ease score of 6.4 (the closer to 100 a score is, the easier it is to read) and a Flesch–Kincaid (FK) Grade level of 23.2 (to be considered truly aphasia‐friendly, the FK Grade should be 5 [37]). Berg et al. [35] put forward an expanded definition of aphasia to emphasise the broad impacts of aphasia on the individual with aphasia and their family and friends. Their definition is 68 words long and has a Flesch Reading ease score of 12.2 and Flesch‐Kincaid Grade level of 17.7, which although an improvement in terms of readability statistics, is still far from being an aphasia‐friendly definition. Whilst these definitions were not intended for a general public audience, they help to illustrate how challenging it is to explain what aphasia is and its impact in a concise and accessible way. In the co‐design workshops, considerable time was spent debating how best to describe aphasia, its impact, and the ways the general public can adapt their communication when they meet a person with aphasia in order to treat them respectfully. A universally accepted and accessible definition of aphasia that can be used consistently for raising awareness of the condition is needed.

Of key importance to PLWA was the need for the awareness campaign to convey that people with aphasia are competent and able to contribute with support [3, 18]. Over the years, many aphasia associations, rehabilitation services, and campaigns have used phrases such as ‘Aphasia is a loss of language, not intelligence’ (e.g., Australian Aphasia Association for Wednesday Without Words in 2018 [38]) or ‘Aphasia is a loss of words, not intellect’ (e.g., [39]). This concept, and how to express it, was discussed in depth in our co‐design workshops, and the team eventually settled on using the word ‘intelligence,’ as they felt this was the most meaningful for a lay audience. Statements such as these, however, are further complicated when Primary Progressive Aphasia (PPA) is considered as other cognitive functions, including memory, planning and executive functions, are affected as the condition advances. In addition, a recent article by Nunn et al. [40] has initiated debate about the need to move toward anti‐ableist practices in aphasia rehabilitation and research. Nunn at al. (2024, p. 2693) argue for the need to ‘avoid comparing aphasia to other disabilities which implies that one disability is better than another,’ and advocate for replacing phrases such as ‘ aphasia is a loss of language not intellect’ with phrases such as ‘people with aphasia know what they want to say and will get their message across with additional time and/or strategies.’ While Nunn and colleagues acknowledge that this recommendation is preliminary and needs further discussion with PLWA, their point is worth considering when moving from this campaign blueprint to implementing the campaign.

### 4.1 Reflections/Critical Perspective

Running international online co‐design workshops on Zoom was a feasible and cost‐effective method of bringing an international team of co‐designers together to work on a plan to try to address a global problem, but it was not without challenges. The greatest challenge was coordination across eight different time zones. To address this challenge, there were three meeting options for each workshop round. A limitation of this approach was that some team members did not have the opportunity to meet each other online during this process and co‐designers with lived experience of aphasia were not represented in every meeting. This arrangement added burden in meeting coordination, preparation of materials, and collation of workshop outcomes. The benefits were an opportunity for in‐depth discussion, time for expressing opinions, and some flexibility for involvement. Where movement did occur between meeting groups, this helped to strengthen rapport between members.

To compensate for not being able to meet altogether, all team members were introduced in the initial round of meetings (using a PowerPoint slide per person and inclusion of a photo), included in all emails, and detailed minutes of each meeting were produced and shared with all team members. To address the principles of transparency, inclusivity, accessibility, and respect, these minutes documented individuals’ thoughts and ideas to enable other team members to understand their perspectives. As a result, these minutes were lengthy and time‐consuming to produce, especially as they were produced in an aphasia‐friendly format [27, 28, 29]. The aphasia‐friendly minutes of the Round 2 meetings were also audio‐recorded to improve accessibility for our co‐designers with aphasia. However, they reported this was not necessary and they were happy to receive a pdf of the aphasia‐friendly minutes and PowerPoint slides only from Round 3 onwards. Co‐designers without aphasia also reflected on appreciating the aphasia‐friendly minutes, finding them easy to read, and one co‐designer reported using this example in a push to make all communications within their organisation more accessible. Another major challenge was that workshops and minutes were in English only.

Reaching an agreement on decisions across the three groups was challenging, and there were still some differences of opinion at the end of the workshop series. Sometimes an individual's views were at odds with the evidence‐based. For example, previous research has found that PLWA want increased awareness about: what aphasia is, the impacts of aphasia, and how to communicate with a person with aphasia [3, 5], but some co‐designers felt that including a call to action about how to respond to a person with aphasia was too much information. This difficulty/consideration has been reported in other PPI literature; for example, in Shiggins et al. [17], where differences of opinion between stakeholders, outcomes at odds with evidence, tension, and a resultant need for compromise were also part of the process. As research practice evolves more towards PPI, this is something that will need further discussion and consideration.

### 4.2 Limitations

This study has several limitations. The co‐design team was relatively small, and workshops were conducted in English. Most co‐designers were from English‐speaking countries, although multilingual speakers from Cyprus, Denmark, India, and Switzerland also participated. Gaps in expertise, particularly advertising and design, remained despite recruitment efforts. Certain perspectives may be underrepresented, particularly those from low‐ and middle‐income countries and people with more severe aphasia who face greater barriers to online participation. However, it should be noted that this co‐design process was informed by the results of a broader research programme that gathered perspectives from 466 participants (131 people with aphasia, 335 professionals) across 40 countries [3, 18]. Co‐designers were purposively selected for diversity in age, sex, country, language, and expertise. To enhance transferability, the team recommended assets representing diverse ethnicities, genders, ages, and abilities, and prioritised simple, aphasia‐friendly language to support cross‐cultural adaptation. Future research should examine how the blueprint performs across diverse settings.

### 4.3 Future Directions

Future directions will require funding to develop and translate campaign assets, engage media and marketing support, and implement the campaign.

## 5 Conclusion

A team of experts spanning seven countries and four continents co‐designed a blueprint for a unified international aphasia awareness campaign and a plan to operationalise it. The ultimate aim of the campaign is to improve the public's understanding of aphasia and the way they interact with people with aphasia, to facilitate greater participation in daily life for those living with aphasia. A multi‐lingual campaign on this scale, targeting the general public, has not been attempted before. A combined effort by the global aphasia community has the potential to amplify the voices of PLWA and bring about a much‐needed societal response to improve the lives of the millions living with aphasia worldwide.

## Author Contributions

**Claire Bennington:** conceptualisation, investigation, funding acquisition, writing – original draft, methodology, writing – review and editing, formal analysis, project administration, data curation, resources. **Ciara Shiggins:** conceptualisation, investigation, writing – review and editing, methodology, supervision, formal analysis. **Jytte Isaksen:** conceptualisation, investigation, writing – review and editing, methodology, supervision, formal analysis. **Emma Beesley:** conceptualisation, investigation, methodology, formal analysis, supervision, writing – review and editing. **Kim Beesley:** conceptualisation, investigation, methodology, writing – review & editing, formal analysis, supervision. **Sarah J. Wallace:** conceptualisation, invstigation, funding acquisition, methodolgy, supervision, writing – review and editing, formal analysis, project administration, data curation. **Jean‐Marie Annoni:** writing – review and editing, investigation. **Jude Czerenkowski:** writing – review and editing, investigation. **Carly Davey:** writing – review and editing, investigation. **Natalie Douglas:** writing – review and editing, investigation. **Alexia Kountouri:** writing – review and editing, investigation. **Caitlin Longman:** writing – review and editing, investigation. **Apoorva Paurinak:** writing – review and editing, investigation. **Sarah Scott:** writing – review and editing, investigation. **Kathryn Shelley:** writing – review and editing, investigation.

## Ethics Statement

Co‐designers did not assume the role of participants in this project and, therefore, did not contribute data. Involvement is distinct from participation in research (as per the NIHR definition (NIHR INVOLVE, n.d.), and co‐designers worked at the ‘collaboration level’ of the spectrum of involvement [41]. Therefore, no formal ethical approval was required for their involvement as co‐designers. However, this research and project were conducted according to the ethical principles (NHMRC, 2016), and in alignment with guidance from Health Consumers Queensland [42], and on the ethics of Patient and Public Involvement (PPI) when working with people with aphasia [21]. People with lived experience of aphasia (people with aphasia and a family member) were paid for their work, in recognition of their skills, expertise, and time. Co‐designers’ contributions are also acknowledged through authorship. No formal consent procedures were required as co‐designers were not acting in the role of participants in this project. However, all co‐designers were invited to join the team, provided with information about the project, voluntarily accepted the invitation, and attended the co‐design sessions. Therefore, consent (active and implied) was provided for involvement.

## Conflicts of Interest

The authors declare no conflicts of interest.

## Supporting information

Supporting File

## Data Availability

Supporting information is available upon reasonable request from the corresponding author (CB).
