# Supplementary material for: Co‐Design of a Unified, International Aphasia Awareness Campaign
Source: Health Expect. 2026 Apr 15;29(2):e70658. doi: 10.1111/hex.70658 (PMC13080883; doi:10.1111/hex.70658)
Supplement: Supplementary file 1 — Supporting File [file HEX-29-e70658-s001.docx]

**Supplementary Material**

**Supplementary File 1:** Advisor *Involvement per PAOLI Framework (Charalambous et al., 2023)*

| **PAOLI Item** | **Examples of involvement of advisors with lived experience** |
| --- | --- |
| Establish collaborations | - Discussed people with lived experience to invite as co-designers onto the co-design team |
| Recruit patients | - N/A |
| Gain informed consent | - N/A as not required |
| Organise induction meetings | - Participated in introductory round of workshops |
| Train patient partners | - N/A |
| Create communication links | - Critically appraised the materials used during the co-design meetings (e.g. aphasia-friendly summaries of results, aphasia-friendly meeting minutes) to ensure communicative accessibility |
| Engage communication partners | - N/A |
| Conceptualise topics | - Contributed to the project aims, design and plan |
| Establish research priorities | - Contributed to the interpretation of results |
| Reach consensus | - Contributed to the interpretation of results and decision-making. The perspectives of people with lived experience took precedence where resolution of differences of opinion was not possible. |
| Work with co-design methods | - Co-facilitated the co-design workshops |
| Develop proposals | - N/A |
| Assist with dissemination of results | - Joint presentation to the Australian Aphasia Association’s 14^th^ National Conference in Perth, November 2023 - Co-authors on presentation to The University of Queensland’s School of Health and Rehabilitation Sciences Research Conference, November 2023 - Suggested a presentation to their local aphasia group - Joint presentation at the Aphasia Access session of the International Aphasia Rehabilitation Conference in Brisbane, July 2024 - Co-authors on a poster presentation at the Academy of Aphasia, Japan, October 2024 - Joint presentation at the consumer event to celebrate the achievements of the Centre of Research Excellence in Aphasia Rehabilitation and Recovery in Melbourne, November 2024 - Co-authors on a poster presentation at the Nordic Aphasia Conference, Sweden, June 2025 - Co-authors on this paper |
| Promote implementation of outcomes | - Have been included in a funding pitch to implement the campaign - Personal story and photographs used to try to raise matched funding in a JustGiving campaign (on Giving page, a paid Facebook ad, and fliers to promote the campaign) . - Have helped to promote the JustGiving Campaign through their networks, on their social media, and distributing flyers in person at international stroke conferences (ANZSO, Tasmania, 2025). |
| Support patient partners and promote self-evaluation | - N/A |
| Monitor progress | - Kept informed of workshop completion each round |
| Assess the impact of the patient involvement | - Beyond this project |

**Supplementary File 2:** *Reporting per GRIPP* ***2*** *(Staniszewska et al., 2017)*

| **Section and topic** | **Item** | **Reported on page no.** |
| --- | --- | --- |
| 1. **Aim** | Report the aim of PPI in the study | p. 5 |
| 1. **Methods** | Provide a clear description of the methods used for PPI in the study | pp. 6-15 |
| 1. **Study results** | Outcomes – Report the results of PPI in the study, including both positive and negative outcomes | pp. 15-26 |
| 1. **Discussion and conclusions** | Outcomes – comment on the extent to which PPI influenced the study overall. Describe positive and negative effects | pp. 26-31 |
| 1. **Reflection / critical perspective** | Comment critically on the study, reflecting on the things that went well and those that did not, so others can learn from this experience | pp. 28-30 |
| PPI = patient and public involvement | | |

**Supplementary File 3:** *Co-design Workshop Rounds*

| **Workshop** | **Focus** |
| --- | --- |
| 1. | - Welcome and plan for workshop - Introductions – sharing our experiences and motivations for the project - About the project:   Co-design  Aim and limits of the project.   - How we will work together - Roles and responsibilities - Ground rules - Tasks before the next workshop - Dates for next round of workshops |
| Familiarisation with the project | **Week 1** – Provide aphasia-friendly video summary of results of international stakeholder perspectives on aphasia awareness (Study 1)  **Week 2** – Provide aphasia-friendly video summary of results of international stakeholder experiences of raising awareness of aphasia (Study 1, Bennington et al., 2024)  **Week 3** - Provide aphasia-friendly video summary of results of international stakeholder priorities for an international aphasia awareness campaign (Study 2, Bennington et al., 2025) |
| 2. | - Welcome and plan for workshop - Review of Round 1 Workshops: - Confirmation of minutes of Round 1. Any comments / questions? - Review of results of Studies 1 and 2. Any comments / questions? - Discuss the desired outcome/s and target audience/s for our campaign - Identify steps / actions to be completed before workshop 3. |
| 3. | - Welcome and plan for workshop - Review of Round 2 Workshops: - Confirmation of minutes of Round 2. Any comments / questions? - Summary of Round 2 discussions re desired outcome/s and target audience/s - Discuss the desired message for our campaign - Identify steps / actions to be completed before Workshop Round 4. |
| 4. | - Welcome and plan for workshop - Review of Round 3 Workshops: - Confirmation of minutes of Round 3. Any comments / questions? - Summary of Round 3 discussions re desired message - Discuss the format and design for our campaign - Identify steps / actions to be completed before Workshop Round 5. |
| 5. | - Welcome and plan for workshop - Review of Round 4 Workshops: - Confirmation of minutes of Round 4. Any comments / questions? - Summary of Round 4 discussions re. format and design - Refine our message (what aphasia is / is not; the impact of aphasia; how to respond to a person with aphasia) and our call/s to action - Discuss how we operationalise the campaign - Identify steps / actions to be completed before final workshop |
| 6. | - Welcome and plan for workshop - Review of Round 5 Workshops: - Confirmation of minutes of Round 5. Any comments / questions? - Summary of Round 5 discussions re. refining our message and how we operationalise the campaign - Any further thoughts / comments? - Summary of all workshops and our decisions - Any further thoughts / comments? - Reflections on this experience. - Next steps - Celebration and thank you |

**Supplementary File 4:** *Sample Aphasia-Friendly Agenda for One of the Meetings in the Second Round of Co-design Workshops in Study 3*


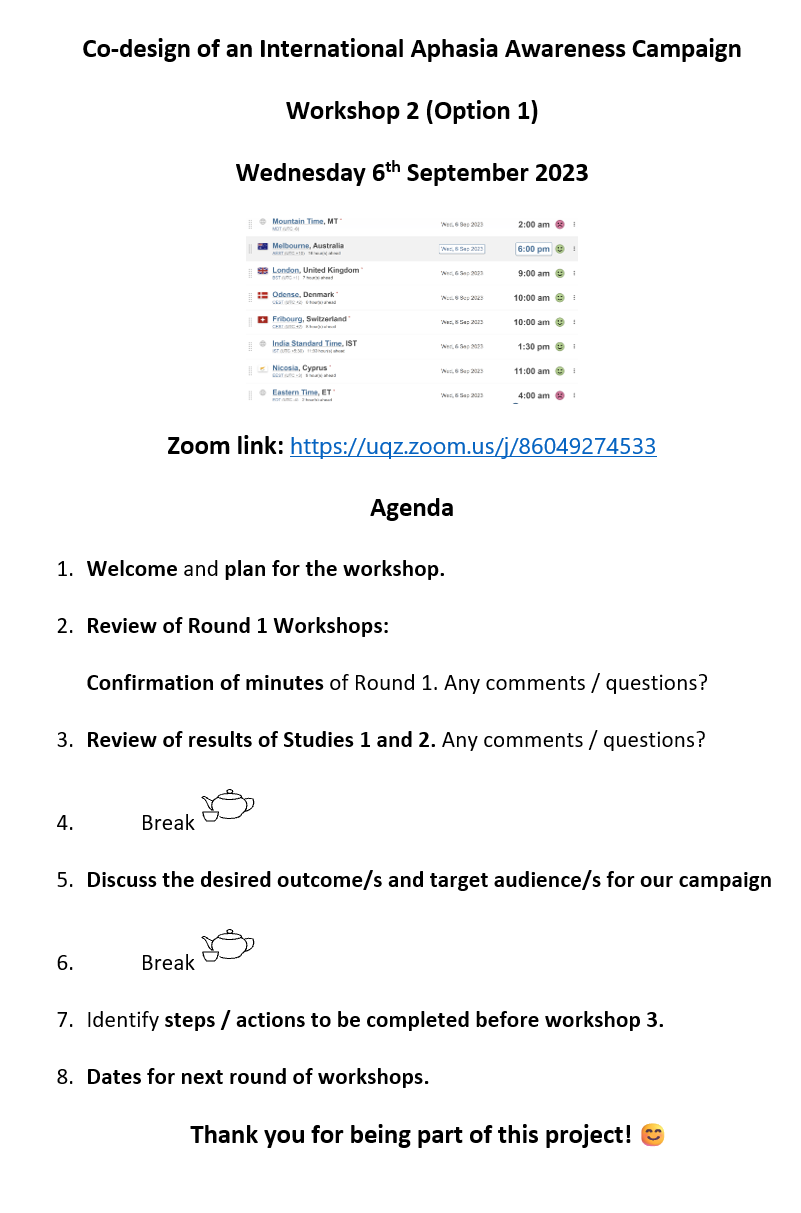


**Supplementary File 5:** *Sample of the PowerPoint slides for Round 2 of the Co-design Workshops*

*
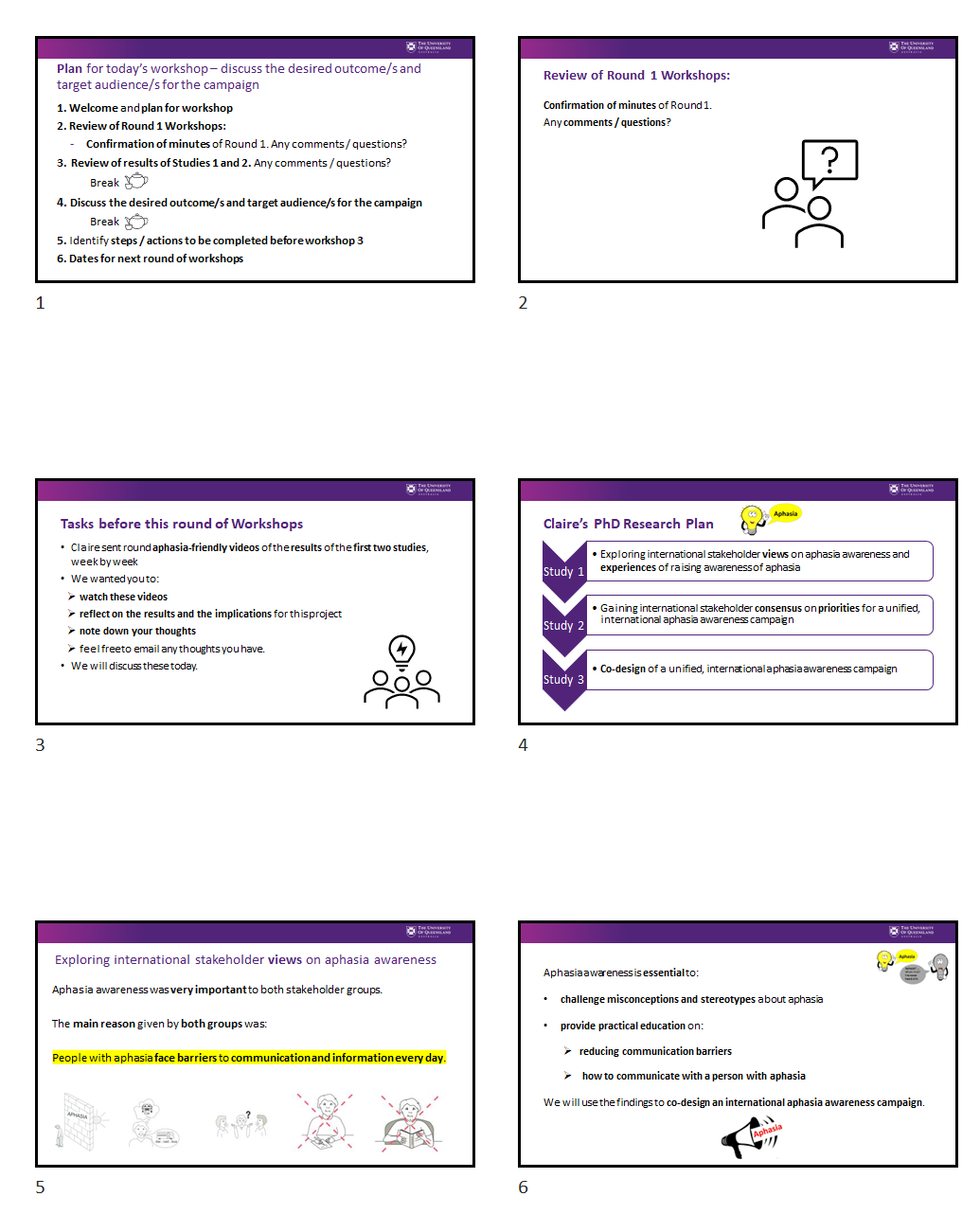
*
